# Polydnavirus Innexins Disrupt Host Cellular Encapsulation and Larval Maturation

**DOI:** 10.3390/v13081621

**Published:** 2021-08-17

**Authors:** Peng Zhang, Matthew Turnbull

**Affiliations:** Department of Biological Sciences, Clemson University, Clemson, SC 29631, USA; pzhang2@g.clemson.edu

**Keywords:** Polydnavirus, innexin, gap junction, parasitoid, encapsulation, bioelectricity

## Abstract

Polydnaviruses are dsDNA viruses associated with endoparasitoid wasps. Delivery of the virus during parasitization of a caterpillar and subsequent virus gene expression is required for production of an amenable environment for parasitoid offspring development. Consequently, understanding of Polydnavirus gene function provides insight into mechanisms of host susceptibility and parasitoid wasp host range. Polydnavirus genes predominantly are arranged in multimember gene families, one of which is the *vinnexins*, which are virus homologues of insect gap junction genes, the *innexins*. Previous studies of *Campoletis sonorensis* Ichnovirus Vinnexins using various heterologous systems have suggested the four encoded members may provide different functionality in the infected caterpillar host. Here, we expressed two of the members, *vnxG* and *vnxQ2*, using recombinant baculoviruses in susceptible host, the caterpillar *Heliothis virescens*. Following intrahemocoelic injections, we observed that >90% of hemocytes (blood cells) were infected, producing recombinant protein. Larvae infected with a *vinnexin*-recombinant baculovirus exhibited significantly reduced molting rates relative to larvae infected with a control recombinant baculovirus and mock-infected larvae. Similarly, larvae infected with *vinnexin*-recombinant baculoviruses were less likely to survive relative to controls and showed reduced ability to encapsulate chromatography beads in an immune assay. In most assays, the VnxG protein was associated with more severe pathology than VnxQ2. Our findings support a role for Vinnexins in CsIV and more broadly Ichnovirus pathology in infected lepidopteran hosts, particularly in disrupting multicellular developmental and immune physiology.

## 1. Introduction

Polydnaviruses (PDVs) are a remarkable virus family. These large dsDNA viruses are obligately and mutualistically associated with specific families of parasitoid wasps, a relationship that generates unusual selection pressures on the viruses. The association of these viruses with their wasp hosts is essential to the ecological and evolutionary success of those wasps. PDVs are a paraphyletic lineage: Bracoviruses (BVs), associated with PDVs are associated with the microgastroid complex of the wasp family Braconidae, while Ichnoviruses (IVs) are associated with Campopleginae and Banchinae wasp subfamilies of the wasp family Ichneumonidae. Despite their evolutionary independence, the different lineages of PDVs exhibit similar genome organization and are integrated as a provirus into the genome of the host wasp. The genome of members of both lineages is comprised by large dsDNA across multiple segments, of which a majority of the sequence is non-coding, while coding sequences cluster into numerous multimember gene families [1,2]. The PDV genome is transmitted vertically as a provirus in the wasp genome, while horizontal transmission occurs across species boundaries: PDV replication and encapsidation precedes oviposition, during which virus is introduced along with egg into the hemocoel of a parasitized insect (typically juvenile lepidopteran, i.e., caterpillar) host. Infection followed by expression of virus genes results in numerous host pathophysiologies, including disruptions of the immune, energy, homeostatic, and endocrine systems, which provide a beneficial environment to the developing wasp [2,3,4]. There is no evidence of PDV replication in the infected secondary host during this period [5].

As virus replication does not occur in the caterpillar host, it generally has been held that PDV genes expressed in that host play a role in generating pathophysiology [3,6,7]. Therefore, the expansion of PDV genes into gene families has been viewed as evidence of selection for manipulation of additional hosts and/or tissues and processes within a host [8,9]. Assigning function to PDV genes then provides insight into the mechanisms by which PDVs manipulate lepidopteran host physiology and by extension those by which the associated parasitoid host range is determined. However, due to the complexities of the association between wasp and virus, it can be difficult to assess the roles of individual genes and/or gene families. Thus, work in both BVs and IVs has sought to elucidate PDV gene function, typically testing sufficiency using in vitro assays or necessity via RNAi during PDV infection [10,11,12,13,14].

Total IV gene expression is associated with multiple host pathologies, such as alterations of the cytoskeleton [15,16,17] and disruption of cell signaling [10]. Among IV genes expressed are members of the *vinnexin* gene family [18]. These genes are virus homologues of insect gap junction genes (*innexins*) and are highly conserved among IVs [19]. The proteins form functional gap junctions in heterologous and cell culture systems [18,20], and in the latter expression of *vinnexins* induces cell membrane depolarization and cytoplasmic alkalization [21]. As Innexins occur and function in hemocytes including during immune responses [22,23,24,25], and gap junction disruption reduces immunocompetence [26], we hypothesize the Vinnexins may interfere in vivo with immune responses, a prediction which has not been thoroughly tested.

To develop a better understanding of the effects of Vinnexins on larval lepidopterans, we used recombinant baculoviruses to express two Vinnexins of the Polydnavirus Campoletis sonorensis Ichnovirus (CsIV), vnxG and vnxQ2, in host *Heliothis virescens* larvae. Our results demonstrate that Vinnexin-expressing caterpillars exhibit stunted development and increased mortality relative to a control recombinant baculovirus. Although Vinnexin expression does not alter total hemocyte numbers relative to the control baculovirus, both Vinnexins are associated with hemocyte membrane depolarization. VnxG and VnxQ2 differentially affect immunocompetence of infected caterpillars, as VnxQ2 reduces cellular immunity, while VnxG decreases consistency of immune response. Thus, the Vinnexins may play a pathological role in IV manipulation of the host and further implicate gap junctions in insect immunity.

## 2. Materials and Methods

### 2.1. Insect Rearing

Third instar *H. virescens* caterpillars were purchased (Benzon Research, Carlisle, PA, USA) and reared at 27 °C. Newly molted 4th instar larvae, staged according to head capsule width [27], were used for experiments, and were maintained at 27 °C after treatment for data collection.

### 2.2. Recombinant Virus Generation and Injection

The recombinant baculoviruses used for in vivo expression of *vnxG-*His and *vnxQ2-*His or control virus, FLAG-*mc4r*, were generated with the Bac-to-Bac vector system (Invitrogen). Details for generation were previously published [21]. Viruses were titered by plaque or end-point dilution assay [28]. For injections, caterpillars were anesthetized by submerging in ddH_2_O for 20 min and then the abdominal skin was sterilized with 70% ethanol and dried with a paper towel. Five microliters Hink’s TNM-FH Insect Medium containing no recombinant virus or 100 pfu recombinant virus (encodes *mc4r*, *vnxG*, *vnxQ2*) were injected laterally by a Hamilton #701 needle. A thin layer of liquid bandage (New-skin, Moberg Pharma North America LLC, Cedar Knolls, NJ, USA) subsequently was applied to the injection site to stop bleeding.

### 2.3. Immunoassays

To collect hemocytes, caterpillars (5 caterpillars per treatment) at 3 days post-treatment were immobilized on ice and bled directly into cold anticoagulant buffer (0.098M NaOH, 0.186M NaCl, 0.017M EDTA, 0.041M Citric acid, pH 4.5). Hemolymph samples were immediately centrifuged for 5 min at 500× *g*, 4 °C. The supernatant was discarded and the hemocyte pellets were gently rinsed twice with cold PBS (pH 7.0). For western blot, hemocytes were resuspended in lysis buffer (25 mM Tris-HCl, pH 7.6; 150 Mm NaCl; 1% NP-40; 0.5% TritonX-100; 0.1% SDS). Equal concentrations of total protein, as determined by Bradford assay, were diluted in 4× loading buffer, incubated for 30 min at 37 °C, separated on 10% polyacrylamide gels (Bio-Rad, Mini-PROTEAN^®^ TGX™ Precast Gels, Hercules, CA, USA), and transferred to PVDF membrane. Blots were probed with rabbit anti-His antibody (Thermo Fisher Scientific, Waltham, MA, USA) at 1:1000 in blocking solution at 4 °C overnight, and then probed with polyclonal donkey anti-rabbit HRP-antibody (Invitrogen) at 1:5000 in blocking solution at room temperature for 1 h. After washing, blots were treated with ECL substrate (Thermo Fisher Scientific), developed, and visualized on X-ray film.

For immunomicroscopy, 10^4^ infected hemocytes were seeded in chamber slide, fixed with 4% formaldehyde (in PBS) at room temperature for 15 min, permeabilized with PBST (PBS + 0.2% TritonX-100) at room temperature for 10 min and blocked with 5% FBS (in PBST) at room temperature for 60 min with washing in PBS between every step. Primary (rabbit anti-His, Thermo Fisher Scientific) and secondary (anti-rabbit Alexa Fluor 594, Jackson ImmunoResearch, West Grove, PA, USA) antibodies were diluted in blocking solution and applied at 1:200 and 1:1000, incubated at 4 °C overnight and room temperature for 1 h, respectively. Images were captured on a Nikon TE2000 epifluorescence microscope with NIS Elements BR 2.3 software.

### 2.4. Caterpillar Survival and Development

Thirty newly molted 4th instar larvae were injected with media or recombinant virus as above, and monitored daily for mortality for one week. Caterpillars were recorded as live, dead, or pupated. Dead caterpillars were removed from diet tray after counting to avoid contamination. Larvae that died during the first three days were considered as mortality due to physical damage, and were removed from analyses. The instar of each caterpillar was recorded at 3 dpi and the proportion of the 4th instar caterpillar was calculated. Each treatment was replicated 3 times.

### 2.5. Hemocyte Count Determination and V_mem_ Measurement

Hemocytes were collected and washed as described above. Hemocyte counts were determined with a Neubauer hemocytometer, with three replicates per treatment. To measure the membrane potential of hemocytes, 10^4^ hemocytes were seeded in a 96-well plate and given 15 min to attach. At that time, Bis-(1.3-Dibutylbarbituric Acid) Trimethine Oxonol (DiBAC4(3)) was added to 1 µg/mL and allowed to incubate for 10 min in the dark. Hemocytes were imaged and analyzed as previously described [21]. In brief, images were captured with a DS-Qi1Mc monochromatic camera on a Nikon TE2000 epifluorescence microscope with FITC filters. Cell intensity was collected by randomly choosing individual cells and manually generating the outline of cells as a region of interest (ROI). Normalized mean intensity (NMI) was calculated for each ROI by normalizing the ROI mean intensity (RMI) to background (BACK) and ambient light (AMB) by the calculation,

NMI = [(RMI-BACK) / (AMB-BACK)](1)

A minimum of 165 cells were analyzed across 3 replicates for each treatment.

### 2.6. Ex Vivo Encapsulation Assay

To test ability of hemocytes to engage in encapsulation, DEAE Sephadex beads were used as targets in ex vivo encapsulation assay. One million hemocytes of from treatment were placed in a 48-well plate coated with a layer of 0.5% sterilized agarose [29]. Ten beads, hydrated and sterilized, were added to each well. After 3 h incubation, beads were collected by gentle pipetting and transferred to a slide and only intact beads were examined for encapsulation. Beads were scored as “Normal encapsulation” if they were completely surrounded by multiple layers of hemocytes. “Abnormal encapsulation” noted both partial encapsulation—75% surface was surrounded by hemocytes and intense encapsulation—the encapsulation was several times as big as the bead itself. Immune assay was repeated 5 times and at least 21 beads were examined for each treatment.

### 2.7. Statistical Analyses and Figures

All statistical analyses were performed in R x64 3.4.3 and Minitab 18. Graphs were generated in DataGraph V4.2.1 (Visual Data Tools Inc., Chapel Hill, NC, USA).

## 3. Results

### 3.1. In Vivo Recombinant Protein Expression

**Figure 1:**
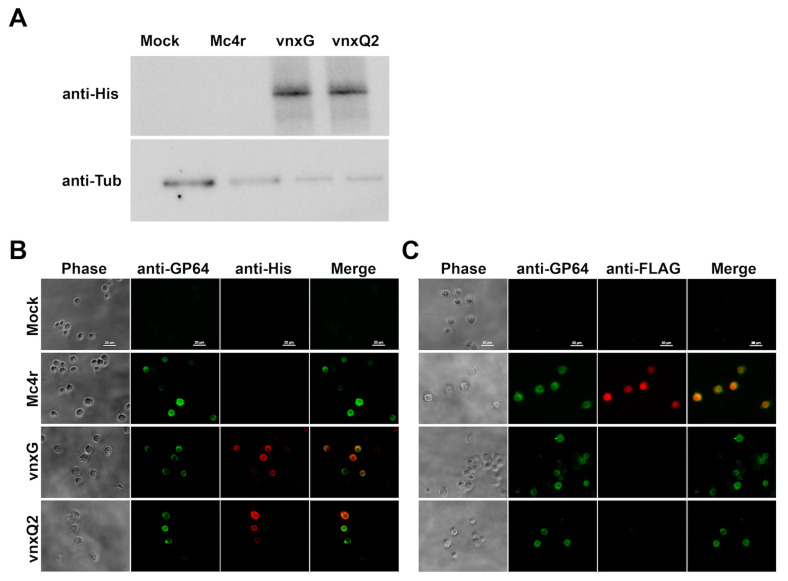
*Vinnexins* are expressed by recombinant baculoviruses in larval *H. virescens* hemocytes. Newly molted 4th instar larvae were injected with 100 pfu recombinant baculoviruses or media and hemocytes isolated 3 dpi. (**A**) Expression of His-tagged *vinnexin* was verified with anti-His western blot. Mock: mock treated; Mc4r: AcMNPV-FLAG-*m*c4r; VnxG: AcMNPV-*vnxG*-His; VnxQ2: AcMNPV-*vnxQ2*-His. Bottom panel is anti-tubulin as loading control. (**B**) Anti-His and anti-GP64 immunomicroscopy was performed to verify Vinnexin expression and AcMNPV infection, respectively. (**C**) Anti-FLAG and Anti-GP64 immunomicroscopy was performed to verify FLAG-Mc4r expression and AcMNPV infection.

We utilized recombinant baculoviruses that were previously characterized [21]. These include two experimental recombinant viruses that encode *Campoletis sonorensis* Ichnovirus (CsIV) *vinnexinG* (*vnxG*) and CsIV *vinnexinQ2* (*vnxQ2*), each with C-terminus 6x-His epitope, and a third recombinant virus encoding a fish melanocortin-4-receptor (Mc4r) with an N-terminus FLAG epitope to serve as control for effects of recombinant virus and overexpression of an exogenous membrane protein. Newly molted 4th instar *H. virescens* larvae were anesthetized and injected with 100 plaque-forming unit (pfu) of recombinant viruses encoding *mc4r*, *vnxG,* or *vnxQ2*, or the same volume of virus-free media. Hemocyte protein was isolated and analyzed by anti-His western blot, which verified recombinant protein expression in hemocyte samples (Figure 1A). We also examined protein expression in hemocytes using epifluorescence immunomicroscopy. Anti-His (VnxG-His, VnxQ2-His; Figure 1B) and anti-FLAG (FLAG-Mc4r; Figure 1C) immunomicroscopy results corresponded to patterns observed Sf9 cells infected with the recombinant viruses [21], and Sf9 and High Five cells transfected with *vinnexin* expression plasmids [30]. Anti-GP64 signal demonstrated AcMNPV infection rates were typically in excess of 90%.

### 3.2. Infection with Vinnexin-Recombinant Baculoviruses Increases Caterpillar Mortality

**Figure 2:**
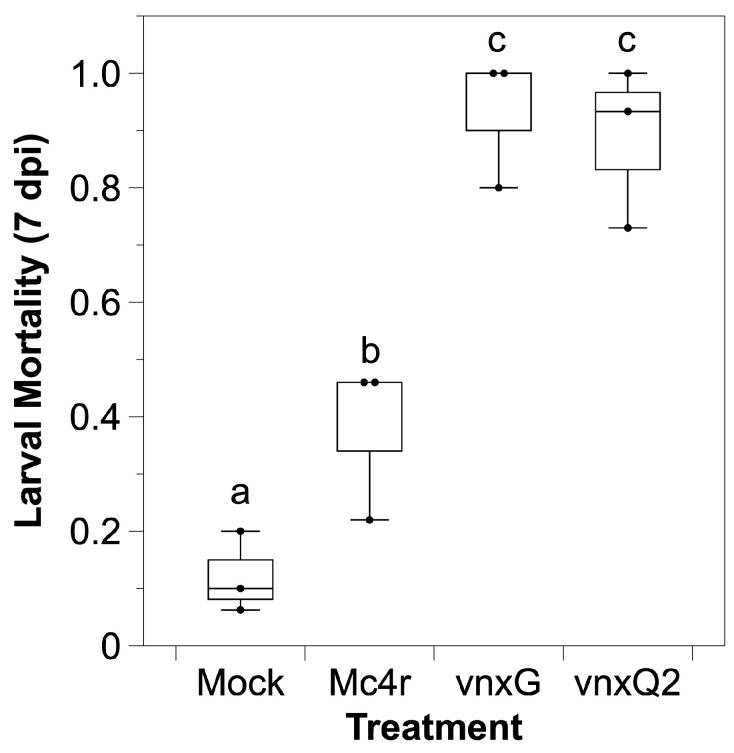
*Vinnexin* expression leads to higher mortality during NPV infection. Early 4th instar larvae were injected with 100 pfu recombinant viruses or media and inspected daily for one week for mortality. Superscript letters indicate significance after Bonferroni correction (α′ = 0.0083).

*H. virescens* is a permissive host to wild type AcMNPV, the baculovirus we used for recombinant virus generation. We tested whether recombinant *vinnexin* expression altered mortality in the presence of an AcMNPV infection. Media or media plus 100 pfu budded control or experimental recombinant AcMNPV was injected intrahemocoelically, and larval mortality was assessed daily for 7 d. Approximately 10% of mock-infected (media) larvae died during the period, while the control Mc4r virus treatment resulted in ~40% mortality and both *vinnexin* recombinants resulted in >85% mortality (Figure 2). Bonferroni correction was conducted ahead of multiple comparison (α′ = 0.0083). AcMNPV-*mc4r* induced significantly higher mortality relative to mock control (*p* < 0.008) that on account of baculovirus vector. Infection with either AcMNPV-*vinnexin*-recombinant virus resulted in significantly greater mortality than the AcMNPV-*mc4r* recombinant virus (*p* < 0.008), although there was no difference between the AcMNPV-*vnxG* and AcMNPV-*vnxQ2* infection results (*p* = 0.43).

### 3.3. Infection with Vinnexin-Recombinant Baculoviruses Slows Development of Host Larvae

**Figure 3:**
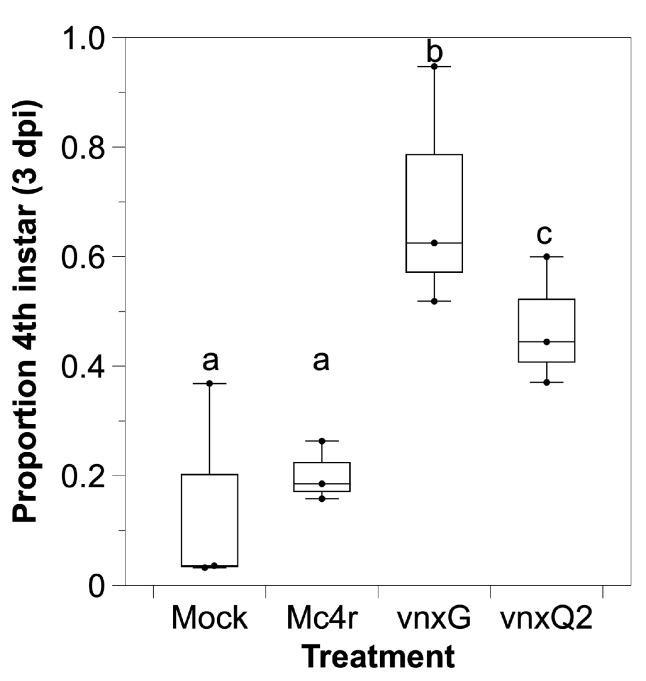
*Vinnexin* expression delays caterpillar development. Mock and recombinant virus infected caterpillars were maintained and observed for 3 days during which developmental stage (4th or 5th instar) was recorded daily. The proportion of 4th instar larvae was calculated and compared. Superscript letters indicate significant difference at *p* < 0.05.

Next, the consequence of *vinnexin* expression on developmental rate was examined. A significant difference in proportion of larvae molting from 4th to 5th instar was observed (Figure 3, χ^2^ (3, *n* = 4) = 58.21, *p* < 0.01). Larvae injected with 100 pfu of control AcMNPV-*mc4r* did not significantly differ from larvae injected with media in the percentage molting from 4th to 5th instar by 3 days post-injection (dpi) (*p* = 0.24). However, both AcMNPV-*vinnexin* differed significantly from both media and AcMNPV-*mc4r* treatments, with significantly more of the AcMNPV-*vnxG*-infected and AcMNPV-*vnxQ2*-infected larvae failing to molt to 5th instar. Furthermore, there was a significant difference between *vnxG*-infected and *vnxQ2*-infected (*p* = 0.02), with significantly fewer AcMNPV-*vnxG*-injected larvae molting.

### 3.4. Vinnexin Expression Has No Effect on Total Hemocyte Count (THC)

**Figure 4:**
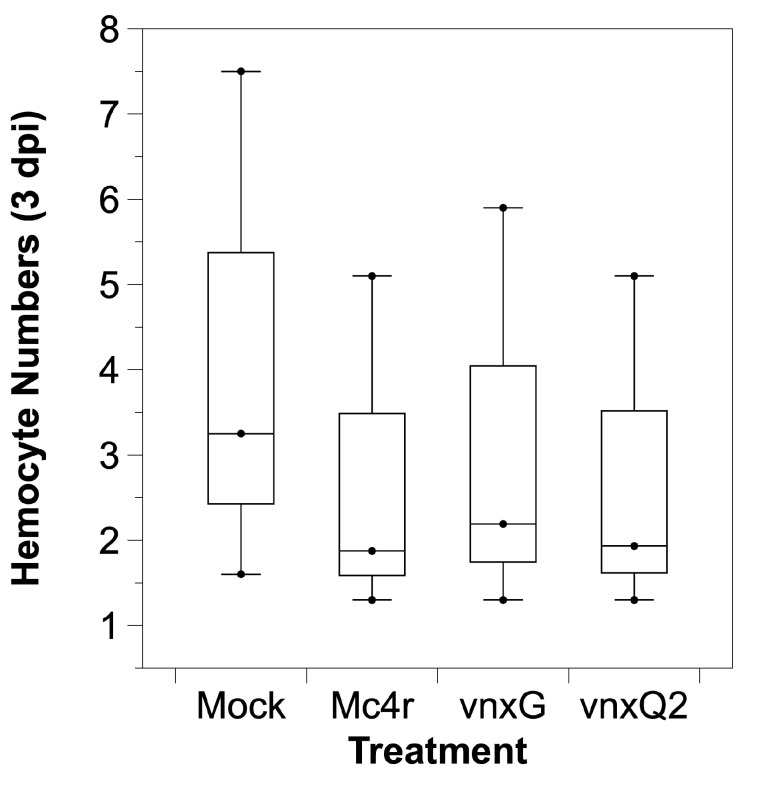
*Vinnexin* expression does not affect total hemocyte count of *H. virescens* larvae. Hemocytes were collected and pooled from three larvae at 3 dpi and counted. The unit of cell count is ten million per milliliter.

Previously, we demonstrated that Sf9 cells infected with AcMNPV-*vnxG* and AcMNPV-*vnxQ2* exhibited a significant reduction in cell number relative to mock-infected and AcMNPV-*mc4r*-infected cells [21]. As a high percentage of hemocytes of larvae injected with the three viruses were GP64 positive (Figure 1B,C), indicating hemocytes were infected with the viruses, we tested whether infection likewise altered hemocyte number in vivo. Although there was a trend to reduced THC in virus infected larvae relative to mock infected, the reduction was not significant (ANOVA, F(3.8) = 0.21, *p* = 0.89) (Figure 4). There was no significant difference between the THC of AcMNPV-*vinnexin*-infected and AcMNPV-*mc4r*-infected larvae (*p* > 0.90) or the AcMNPV-*vnxG*-infected and AcMNPV-*vnxQ2*-infected larvae (*p* > 0.99).

### 3.5. Vinnexin Expression Depolarizes Hemocyte Cell Membrane

**Figure 5:**
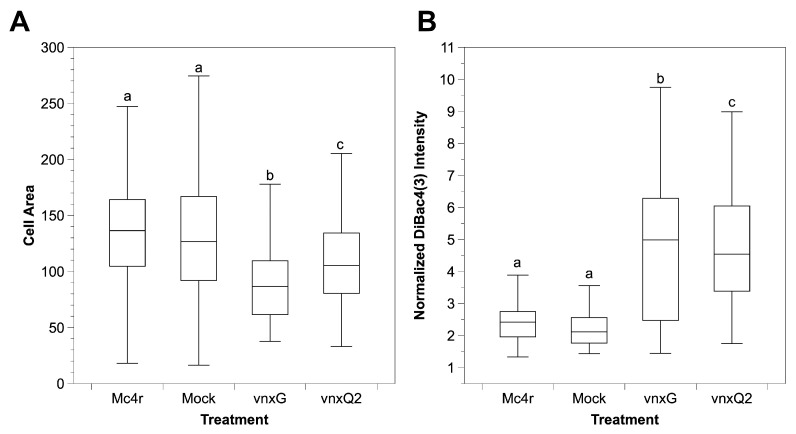
*Vinnexin* expression causes depolarization of hemocytes. Hemocytes of each treatment were incubated with media containing 1 μg/mL DiBAC4(3) at 3 dpi. (**A**) Area and (**B**) normalized DiBac4(3) intensity of isolated hemocytes. Superscript letters indicate significant difference at *p* < 0.05.

Cell membranes of Sf9 cells infected with AcMNPV-*vnxG* or AcMNPV-*vnxQ2* were found to be significantly depolarized relative to controls in a previous study [21]. To test for this phenomenon in vivo, we isolated hemocytes from mock- and recombinant AcMNPV-infected larvae and incubated them with the membrane potential sensitive dye DiBAC4(3). Cells were manually outlined, and area and normalized intensity determined. Cell area differed significantly between treatments (ANOVA, F(3.1116) = 36.53, *p* < 0.01) (Figure 5A). While AcMNPV-*mc4r* infection did not alter area relative to mock (*p* = 0.99), hemocytes from both AcMNPV-*vnxG* (*p* < 0.01) and AcMNPV-*vnxQ2* (*p* < 0.01) infected larvae were significantly smaller than those of AcMNPV-*mc4r* control. Hemocytes isolated from AcMNPV-*vnxG*-infected larvae were significantly smaller than those from the AcMNPV-*vnxQ2*-infected larvae (*p* < 0.01).

Normalized DiBAC4(3) intensity (i.e., V_mem_) was significantly affected by recombinant virus infections relative to mock (ANOVA, F(3.1116) = 121.6, *p* < 0.01) (Figure 5B). AcMNPV-*mc4r* infection significantly increased normalized DiBAC4(3) relative to mock (*p* = 0.01), while AcMNPV-*vnxG* (*p* < 0.01) and AcMNPV-*vnxQ2* (*p* < 0.01) significantly increased normalized DiBAC4(3) values in comparison to the AcMNPV-*mc4r* control. Hemocytes from AcMNPV-*vnxG* infected individuals exhibited significantly higher normalized DiBAC4(3) values than those from AcMNPV-*vnxQ2* (*p* < 0.01). Thus, our data indicate that infection with a *vinnexin*-expressing AcMNPV induces membrane depolarization in hemocytes, similar to our previous findings in Sf9 cells [21].

### 3.6. Vinnexin Expression Disrupts Hemocyte Encapsulation Function

**Figure 6:**
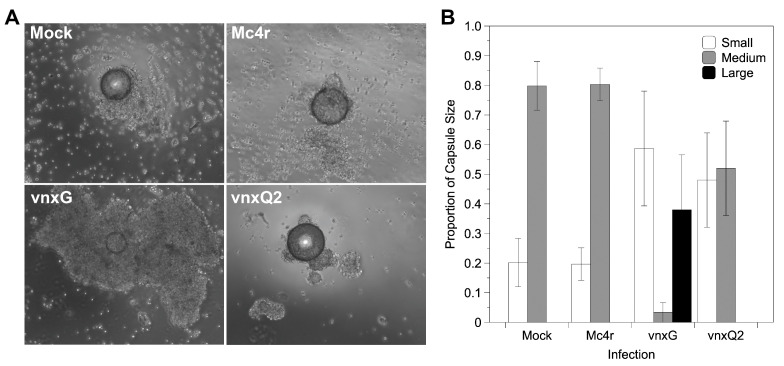
*Vinnexin* expression disturbs hemocyte encapsulation function. Isolated hemocytes cultured with chromatography beads were imaged for formation of cellular capsules. (**A**) Representative micrographs of encapsulation results for each treatment. (**B**) Proportion of encapsulation shown by hemocytes of each treatment according to a 1–3 scale (see text); bars and variance represent mean and s.e.m., respectively.

The impact of *vinnexin* expression on larval immunocompetence was assessed using an ex vivo encapsulation assay. Hemocytes were isolated from mock or recombinant AcMNPV- infected larvae 3 dpi and challenged with chromatography beads in an agarose-lined well. Hemocytes from mock- and AcMNPV-*mc4r*-injected individuals typically encapsulated beads: >80% of beads were surrounded by several layers of hemocytes, forming a regular shaped capsule (2 on the scale used), while <20% of beads showed irregular or no encapsulation (1 on scale) (Figure 6A,B). Hemocytes from AcMNPV-*vnxQ2*-injected individuals failed to encapsulate nearly 50% of the presented beads. Intriguingly, hemocytes from AcMNPV-*vnxG*-injected individuals seldomly formed regular capsules (6%), instead failing to form a capsule or forming a partial one (1 on scale) around >55% of their target beads, and forming larger and more intensive capsules around the remaining beads (3 on scale).

For statistical purposes, partial and intensive encapsulations were classified as “abnormal encapsulation” as opposed to “normal encapsulation”. Abnormal encapsulation rate was calculated and compared among the four treatments (χ^2^ = 40.637, df = 3, *p* < 0.05). Multiple comparison was conducted after Bonferroni correction (α′ = 0.0083). While the two controls did not differ significantly in percentage of beads encapsulated (χ^2^ = 0, *p* = 1.0), AcMNPV-*vnxG* differed significantly from both mock treated (χ^2^ = 30.767, *p* < 0.0083) and AcMNPV-*mc4r* (χ^2^ = 30.767, *p* < 0.008). Although AcMNPV-*vnxQ2* had more partial encapsulation, the proportion was not significantly different from either mock (χ^2^ = 3.857, *p* = 0.05) or AcMNPV-*mc4r* (χ^2^ = 3.857, *p* = 0.05).

## 4. Discussion

Genome duplication in viruses is predicted to result in loss or novel functions where it provides for selective advantage [31]. The relationship of PDVs to their host wasp has long suggested to researchers that expansion of the virus gene families should result in altered host susceptibility [32,33]. Previous studies with PDVs have supported that duplication results in functional novelty or sub-functionalization: there are transcriptional differences seen between pathogenic hosts for a particular PDV, such as with HdIV [34], DfIV [35] and CsIV [33], and protein expression differences between host tissues, such as CcIV *vankyrin* [36] and MdBV PTPases [37], implying evolutionarily significant sub-functionalization. However, it is important to empirically test for potential sub-functionalization in an appropriate physiological context to determine potential contribution of the individual gene family members to host suitability. To that end, we tested two CsIV Vinnexins for their pathogenic contribution in a host, *H. virescens*, particularly in light of previously characterized Vinnexin pathophysiology noted in heterologous systems [21,26].

We utilized recombinant baculoviruses to drive transgene expression in caterpillar hosts. We observed cellular localization (Figure 1) reminiscent of VnxQ2 in CsIV-infected caterpillars [18], as well as VnxG and VnxQ2 expression in cell culture [21,26,30]. We observed increased mortality (Figure 2) and reduced molting (Figure 3) of AcMNPV-*vinnexin*-infected larvae over the mock and recombinant virus controls. Previous studies with injections of purified CsIV have described increased mortality and molt inhibition [38]. Developmental inhibition has been linked to alterations in Juvenile Hormone/Ecdysone titers [39], which in turn have been associated with reduced prothoracicotropic gland size and activity [40]. CsIV *vankyrin* expression in *Drosophila melanogaster* using the GAL4/UAS system indicates that single members of that gene family are sufficient to induce prothoracic gland cell dysfunction [41]. We have not tested *vinnexin* expression or protein effect on endocrine gland function or circulating hormone titers. However, while the Vinnexins have inconsistent effects on cell number (Figure 4; ref. [21,26]), it seems more likely that the molt pathology associated with them is general rather than targeted for the developmental system, as transcription of all four *Vinnexins* is detected in all tested tissues in infected *H. virescens* [18]. To that end, we suspect that the developmental delay observed here is due to cryptic cell death and loss of homeostasis, rather than pathology specific to the molting axis.

We also recapitulated previous in vitro observations of Vinnexin-specific alteration of membrane potential. Previously, we demonstrated that CsIV VnxG and VnxQ2 induce membrane depolarization and cytoplasmic alkalization in expressing Sf9 cells [21]. Here, we found that hemocytes isolated from individuals infected with the AcMNPV-*vnxG* were depolarized relative to those isolated from AcMNPV-*vnxQ2* infected individuals, which in turn were depolarized relative to mock and control recombinant-infected hemocytes (Figure 5). Intriguingly, little examination of the relationship between hemocyte function and membrane potential, as well as with endogenous and exogenous electrical fields, has been performed in insects. Caterpillar hemocytes undergo depolarization during immune stimulation [42], although the mechanism(s) and significance of this phenomenon are unknown. *Drosophila melanogaster* hemocyte calcium transients are required for orientation and migration, and disruption by a parasitoid venom component reduces cellular immunity [43]. While circumstances suggest that the Vinnexin-induced depolarization is important to hemocyte pathology, the mechanisms underlying it are currently unknown [21], and both mechanism and implications are under study.

VnxG and VnxQ2 both alter immune responses in expressing caterpillars relative to mock and recombinant virus controls (Figure 6). Intriguingly, while VnxG disrupts encapsulation resulting in under- or over-formation of capsules, hemocytes from AcMNPV-*vnxQ2*-infected individuals typically failed to form a capsule. It is possible that the two proteins differ due to inability of one to interact with *H. virescens* cellular machinery. While multiple virus hosts were not tested here, previous work has demonstrated consistency in pathological effect across numerous heterologous systems: VnxG forms stronger, more reliable gap junctions in *Xenopus* oocytes [18,20], its expression is embryonic lethal in *D. melanogaster* embryos while VnxQ2 is not [26] and VnxG is more strongly alkalizing and depolarizing in the *Spodoptera frugiperda*-derived Sf9 cell line [21]. Reduced pathogenicity is observed with VnxQ2 in these different systems, suggesting that while VnxQ2 is competent for interacting with host cells in a consistent fashion, it does so to a lesser effect than VnxG. The significance of this difference is currently unclear, but may aid in elucidating the consequence of gene family diversification during PDV evolution.

Previous studies observed the presence of gap junctions [25,44] and occurrence of electrical conductance [45] in hemocytic capsules, suggesting gap junctions play a role in the encapsulation immune response. Blockage of gap junctional intercellular communication via intrahemocoelic injection of carbenoxolone reduces capsule formation [26], supporting a role for gap junctional intercellular communication in capsule morphogenesis. Together, these data suggest that Innexins are crucial to encapsulation, in part via gap junctional intercellular communication. Our findings that in vivo expression of Vinnexins, which alter Innexin-based gap junctional communication [20], reduce encapsulation (Figure 6) further suggests that intercellular communication via gap junctions is necessary for capsule morphogenesis. However, the mechanism by which Vinnexins may disrupt capsule formation (such as alteration of intercellular communication, ablation of intercellular molecular gradients, or disruption of other intercellular junctions) currently remains unclear.

We performed descriptive and functional assays representing multiple physiological systems within the host *H. virescens* for two members of the CsIV *vinnexin* gene family. We observed significant variation in functionality between VnxG and VnxQ2, which is consistent with direction when compared to other tested systems. Our results thus suggest that the Vinnexins interact consistently and reliably between systems across hosts. However, it remains to be tested whether Vinnexins equally affect different hosts, which will be essential to address their potential role in evolution of host range and physiological susceptibility.

## Data Availability

Not applicable.
